# The inner state differences of preterm birth rates in Brazil: a time series study

**DOI:** 10.1186/s12889-016-3087-9

**Published:** 2016-05-17

**Authors:** Rosana Rosseto de Oliveira, Emiliana Cristina Melo, Elizabeth Fujimori, Thais Aidar de Freitas Mathias

**Affiliations:** Graduate Nursing Program, State University of Maringá, Paraná, Brazil; Health Department, Northern Paraná State University, Paraná, Brazil; School of Nursing, Department of Nursing in Community Healthcare, University of São Paulo, São Paulo, Brazil

**Keywords:** Preterm birth, Health information system, Time series studies, Health statistics, Maternal and child health, Epidemiology

## Abstract

**Background:**

Preterm birth is a serious public health problem, as it is linked to high rates of neonatal and child morbidity and mortality. The prevalence of premature births has increased worldwide, with regional differences. The objective of this study was to analyze the trend of preterm births in the state of Paraná, Brazil, according to Macro-regional and Regional Health Offices (RHOs).

**Methods:**

This is an ecological time series study using preterm births records from the national live birth registry system of Brazil’s National Health Service - Live Birth Information System (Sinasc), for residents of the state of Paraná, Brazil, between 2000 and 2013. The preterm birth rates was calculated on a yearly basis and grouped into three-year periods (2000–2002, 2003–2005, 2006–2008, 2009–2011) and one two-year period (2012–2013), according to gestational age and mother’s Regional Health Office of residence. The polynomial regression model was used for trend analysis.

**Results:**

The predominance of preterm birth rate increased from 6.8 % in 2000 to 10.5 % in 2013, with an average increase of 0.20 % per year (r^2^ = 0.89), and a greater share of moderate preterm births (32 to <37 weeks), which increased from 5.8 % to 9 %. The same pattern was observed for all Macro-regional Health Offices, with highlight to the Northern Macro-Regional Office, which showed the highest average rate of prematurity and average annual growth during that period (7.55 % and 0.35 %, respectively). The trend analysis of preterm birth rates according to RHO showed a growing trend for almost all RHOs – except for the 7^th^ RHO where a declining trend was observed (−0.95 a year); and in the 20^th^, 21^st^ and 22^nd^ RHOs which remained unchanged. In the last three-year of the study period (2011–2013), no RHO showed preterm birth rates below 7.3 % or prevalence of moderate preterm birth below 9.4 %.

**Conclusions:**

The results show an increase in preterm births with differences among Macro-regional and RHOs, which indicate the need to improve actions during the prenatal period according to the specificities of each region.

## Background

Preterm birth, defined as a birth that occurs prior to the 37^th^ week of gestation, is a serious public health problem [1], as it is linked to perinatal mortality, as well as several health complications including respiratory, gastrointestinal immunological, auditory, visual, cognitive, behavioral and growth problems [2]. It is also linked to higher rates of mortality among children (1 to 5 years of age) as well as young adults (18 to 36 years of age), which demonstrates that the consequences of preterm birth can have lifelong effects [3, 4].

With the decline in mortality among the 1–59 month age range, deaths during the first 28 days (neonatal deaths) came to represent a growing share. The main causes of neonatal deaths in 2010 were complications from preterm birth, as well as complications during delivery and pneumonia/septicemia [5]. The World Health Organization report shows that preterm birth is increasing in most countries, and it has become the main cause of death for children under five years of age [3]. Furthermore, the chances of long-term survival for the 15 million babies worldwide born prematurely each year vary widely depending on where they are born [6]. South Asia and sub-Saharan Africa represent over 60 % of the world’s preterm births and concentrate more than 80 % of the 1.1 million deaths due to complications of prematurity [6].

Although Brazil has exceed the Millennium Goals set by the United Nations, with a reduction in child mortality to 15.7 in 2015, it is still among the 10 countries with the highest rates of preterm births worldwide [2]. A nationwide study using data from 2011–2012 found an 11.3 % rate of preterm births in Brazil [7]. A review of population-based studies evidenced an increase on rates of preterm births, which rose from 3.4 % to 15 % in the South and Southeast regions between 1978 and 2004, and from 3.8 % to10.2 % in the Northeast region between 1984 and 1998 [8]. In the state of Paraná, located in the South region of the country, the rate of preterm births rose from 6.2 % in 2005 to 11.9 % in 2012 [9].

Considering that there are regional differences in preterm birth rates [10] it is justified performing studies that analyze its trends and distribution in the countryside of the states in order to contribute for the implementation of public policies and promotion and prevention actions at local level to reduce the incidence of preterm births and consequently neonatal morbidity and mortality. Thus, the objective of this study is to analyze the trend of preterm births in the state of Paraná, from the Macro-regional and Regional Health Offices (RHOs), in the period between 2000 and 2013.

## Methods

This is an ecological, time series study of preterm births recorded in the Live Birth National Information System (Sinasc) for residents of the state of Paraná, Brazil, from 2000 to 2013. Paraná is considered the fifth largest economy in Brazil and is located in the country’s Southern region with an area of 199,880 km^2^ [11]. It has 399 municipalities, grouped into 22 RHOs and four Macro-regional Health Offices: Center-East-South, West, Northwest and North. A Regional Health Office is an administrative division of the state health department, characterized by supporting municipalities in all health areas impacting the management of regional issues [12].

To analyze the trend of preterm births, data from 2000 to 2012 were acquired from Sinasc, and 2013 data were provided by the 15^th^ State Regional Health Office, because they were not yet available online at the National Health Service database (Datasus). Sinasc is a nationwide information system introduced in Brazil in 1990 with the purpose of understanding the epidemiological profile of all live births which makes possible not only the formulation of health diagnoses but also promotes surveillance and monitoring of newborns, with evaluation of health actions as well as the opportunity of population based analyzes on live births [13].

The period between 2000 and 2013 was defined after exploring the completion of the variables listed on Sinasc, by analyzing the rate of undeclared variables, that is, variables that were ignored or left blank. To determine the quality of the database and its potential it was used a scale which was adapted to fit the reality of the satisfactory quality of information filled into Sinasc in the state of Paraná: *excellent*, a percentage of undeclared variables below 1 %; *good*, between 1 % and 2.99 %; *regular*, from 3 % to 6.99 %; and *poor* quality when the percentage of undeclared variables is equal or greater than 7 % [14]. The year with the worst rate of undeclared data was 1999, in contrast to 2013 when all variables were considered as having excellent rates of completion. Accordingly, it was decided not to include data from the year 1999.

The rates of preterm births (<37 weeks) were analyzed and calculated in relation to total live births. Preterm birth rates were calculated according to gestational age (GA): under 28 weeks (extreme prematurity); from 28 to <32 weeks (very premature); and from 32 to <37 weeks (moderate preterm birth), and mother’s place of residence (Regional Health Office and Macro-regional Health Office) [6].

The preterm birth rates were calculated year-to-year and also classified into gestational week intervals for the trend analysis, and grouped into three-year periods (2000–2002, 2003–2005, 2006–2008, 2009–2011) and one two-year period (2012–2013) for being represented in graphics. Thematic maps were drawn for a first and last three-year periods (2000–2002 and 2011–2013), using greyscale, with lighter colors for lower rates and darker colors for higher rates. We opted for the use of three-year periods, due the stratification into gestational week intervals, which can lead to low number of events, likely to cause problems of statistical significance because of the occurrence of random fluctuations in the number of premature births.

The polynomial regression statistical model was used for the trend analysis in which preterm birth rates were regarded as dependent variable (y) and the years of the study the independent variable (x). The ‘year’ variable was transformed into the year-centered variable (x-2006) and the series were mitigated using a three-point moving average. The linear (y = β_0_ + β_1_x_1_), quadratic (y = β_0_ + β_1_x_1_ + β_2_x_2_), and cubic polynomial regression models (y = β_0_ + β_1_x_1_ + β_2_x_2_ + β_3_x_3_) were tested. Any trend whose estimated model reached a *p-value* <0.05 was considered significant. In order to select the best model, it was also considered the analyzes of the scatter plot, the value of the coefficient of determination (r^2^) and residual analysis (assumption of real homoscedasticity). When all criteria were significant for more than one model and the coefficient of determination was similar, the simpler model was chosen. All analyses were carried out using SPSS software, version 20.1.

The research project was approved by the Standing Committee for Ethics in Research of the Paraná State Health Secretariat/Workers Hospital (decision 406,927/2013). All data were obtained from public databases (http://datasus.saude.gov.br/). All data were anonymized.

## Results

**Fig. 1:**
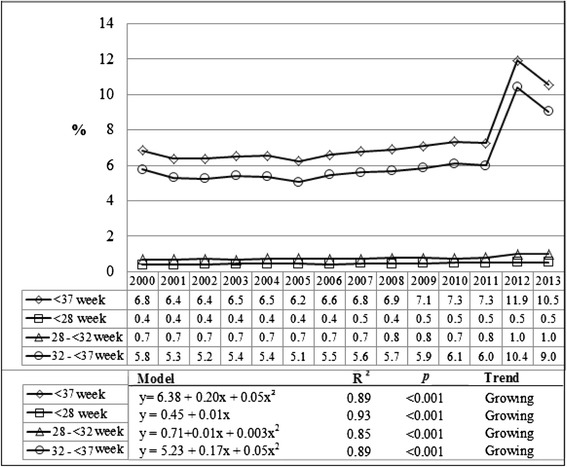
Preterm birth rates trends, according to gestational age. Paraná, Brazil, 2000–2013

The study analyzed 2,219,204 births by mothers residing in the state of Paraná, in the period between 2000 and 2013. Out of those, 163,337 (7.36 %) were preterm births, rising from 6.8 % in 2000 to 10.5 % in 2013, with a higher share of moderate preterm births (32 to <37 weeks), which went from 5.8 % in 2000 to 9 % in 2013 (Fig. 1).

Polynomial regression analysis for the state of Paraná showed a growing trend for preterm birth, with an average yearly increase of 0.20 % (r^2^ = 0.89). The highest average increase occurred for moderate preterm birth (0.17 % per year), with a high coefficient of determination of the model (r^2^ = 0.89) (Fig. 1).

**Fig. 2:**
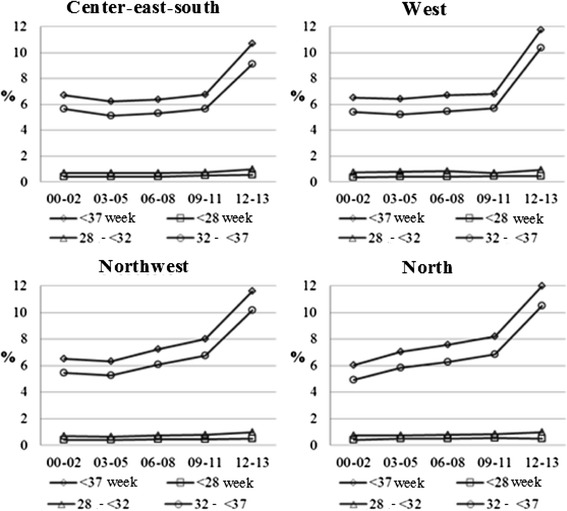
Preterm birth rates, according to gestational age and Macro-regional Health Offices. Paraná, Brazil, 2000–2013

Analysis of preterm birth rates according to gestational age intervals showed a prevalence of prematurity for all Macro-regional Health Offices, especially at the end of the period and the last two-year period (2012–2013). The highest preterm birth rate was observed in 2012–2013 (12.0 %) at the North Macro-regional Health Office, which showed continued, expressive growth throughout the entire period. In contrast, the moderate preterm birth rate at the Center-East-South Macro-regional Health Office decreased slightly during the three-year period from 2003 to 2005 (Fig. 2).

Table 1 shows that all Macro-regional Health Offices had a growing trend for preterm birth rates in all gestational age intervals – except for moderate preterm birth rates at the Center-East-South Macro-regional Health Office, that showed a second-order model with a reduced trend for moderate preterm births in the first part of the period (until 2005), and growth in later years; and the very premature birth (28 to <32 weeks) at the West Macro-regional Health Office, which remained constant (*p* = 0.558). The highest average rate of preterm births and mean annual growth was found for North Macro-regional Health Office, averaging 7.55 % of preterm births and an annual growth of 0.35 %. For its part, the Center-East-South Macro-regional Health Office showed the lowest mean prevalence for the period (6.06 %) and lower mean annual growth (0.13 %) (Table 1).

**Table 1:** Linear regression models for preterm birth rates, according to gestational age and Macro-regional. Paraná, Brazil, 2000-2013

Center-east-south	Model	R^2^	*p*	Trend
<37 weeks	y = 6.06 + 0.13x + 0.06x^2^	0.89	<0.001	Growing
<28 weeks	y = 0.42 + 0.01x + 0.002x^2^	0.91	<0.001	Growing
28 - <32	y = 0.67 + 0.01x + 0.004x^2^	0.90	<0.001	Growing
32 - <37	y = 4.98 + 0.11x + 0.06x^2^	0.89	<0.001	Growing
West				
<37 weeks	y = 6.26 + 0.17x + 0.06x^2^	0.76	0.001	Growing
<28 weeks	y = 0.41 + 0.01x	0.64	0.002	Growing
28 - <32	y = 0.78-0.003x	0.04	0.558	Constant
32 - <37	y = 5.06 + 0.16x + 0.06x^2^	0.79	<0.001	Growing
Northeast				
<37 weeks	y = 6.70 + 0.27x + 0.05x^2^	0.96	<0.001	Growing
<28 weeks	y = 0.44 + 0.01x	0.92	<0.001	Growing
28 - <32	y = 0.71 + 0.02x	0.88	<0.001	Growing
32 - <37	y = 6.14 + 0.29x	0.75	<0.001	Growing
North				
<37 weeks	y = 7.55 + 0.35x	0.86	<0.001	Growing
<28 weeks	y = 0.48 + 0.01x	0.70	0.001	Growing
28 - <32	y = 0.79 + 0.02x	0.76	<0.001	Growing
32 - <37	y = 6.28 + 0.33x	0.85	<0.001	Growing

The polynomial regression analysis of preterm births according to Regional Health Office showed a growing trend for almost all RHOs, with the exception of the 7^th^ RHO - Pato Branco, which showed a declining trend (−0.95 a year). At the 11^th^ RHO - Campo Mourão, there was a reduction in preterm births at the start of the period (year 2005) with a subsequent increase, whereas at the 1^st^ RHO - Paranaguá, 20^th^ RHO - Toledo, 21^st^ RHO - Telêmaco Borba and 22^nd^ RHO - Ivaiporã the premature rates remained constant (Table 2).

**Table 2:** Linear regression models for preterm birth rates, according to Regional Health Office. Paraná, Brazil, 2000-2013

Center-east-south	Model	R^2^	*p*	Trend
1^st^ Paranaguá	y = 6.92 + 0.10x	0.11	0.283	Constant
2^nd^ Metropolitana	y = 7.00 + 0.22x	0.70	0.001	Growing
3^rd^ Ponta Grossa	y = 5.19 + 0.16x + 0.05x^2^	0.69	0.002	Growing
4^th^ Irati	y = 4.95 + 0.40x + 0.07x^2^	0.97	<0.001	Growing
5^th^ Guarapuava	y = 4.60 + 0.19x + 0.09x^2^	0.83	<0.001	Growing
6^th^ União da Vitória	y = 6.77 + 0.30x	0.74	<0.001	Growing
7^th^ Pato Branco	y = 6.48-0.95x + 0.27x^2^	0.86	<0.001	Declining
21^st^ Telêmaco Borba	y = 5.87 + 0.18x	0.18	0.163	Constant
West				
8^th^ Francisco Beltrão	y = 6.21 + 0.26x	0.73	<0.001	Growing
9^th^ Foz do Iguaçu	y = 6.19 + 0.23x	0.57	0.004	Growing
10^th^ Cascavel	y = 6.93 + 0.12x + 0.06x^2^	0.71	0.001	Growing
20^th^ Toledo	y = 7.59 + 0.20x	0.21	0.130	Constant
Northeast				
11^th^ Campo Mourão	y = 5.87-0.29x + 0.07x^2^ + 0.02x^3^	0.89	<0.001	Declining/Growing
12^th^ Umuarama	y = 8.02 + 0.09x	0.56	0.005	Growing
13^th^ Cianorte	y = 5.97 + 0.30x	0.75	<0.001	Growing
14^th^ Paranavaí	y = 5.53 + 0.34x + 0.07x^2^	0.93	<0.001	Growing
15^th^ Maringá	y = 7.90 + 0.47x	0.91	<0.001	Growing
North				
16^th^ Apucarana	y = 7.00 + 0.85x	0.89	<0.001	Growing
17^th^ Londrina	y = 8.59 + 0.34x	0.92	<0.001	Growing
18^th^ Cornélio Procópio	y = 6.22 + 0.18x + 0.07x^2^	0.76	0.001	Growing
19^th^ Jacarezinho	y = 5.26 + 0.30x	0.60	0.003	Growing
22^nd^ Ivaiporã	y = 7.96 + 0.11x	0.24	0.110	Constant

**Fig. 3:**
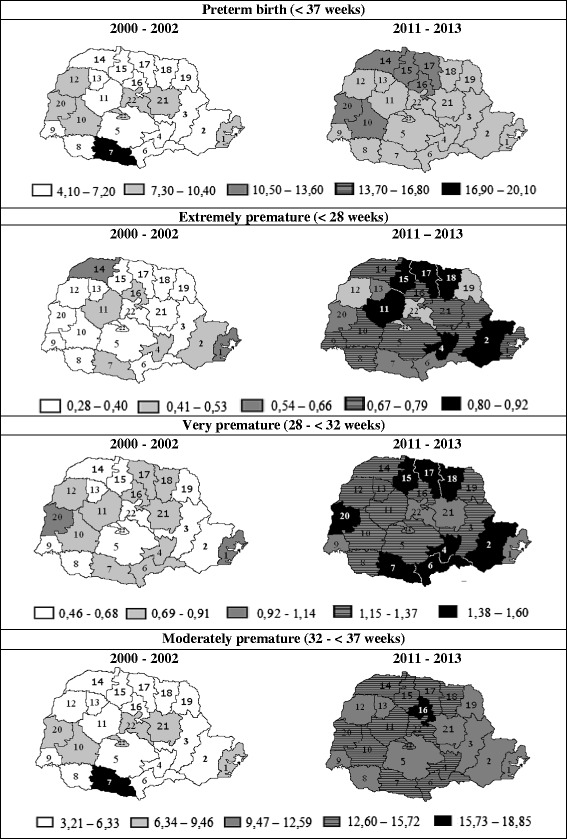
Preterm birth rates, according to gestational age and Regional Health Office. Paraná, 2000–2002 and 2011–2013

The Fig. 3 represents the thematic map of the state of Paraná by RHOs and shows the distribution of preterm birth rates according to gestational age, at the first and the last three-year period. Upon visual inspection, it is observed that the highest rates were found in the last three-year period, when no RHO had a preterm birth rate below 7.3 % or moderate preterm birth (32 to <37 weeks) below 9.4 %. The prevalence of extremely premature births rates (<28 weeks) doubled between the first to the last three-year period at the 15^th^ RHO - Maringá, 17^th^ RHO - Londrina and 18^th^ RHO - Cornélio Procópio, in the same manner as the prevalence of very premature births doubled at the 15^th^ RHO - Maringá and 2^nd^ RHO - Metropolitan (Fig. 3).

Noteworthy is the case of the 7^th^ RHO - Pato Branco, which had a high rate of preterm births (20.1 %) during the three-year period from 2000 to 2002, with a subsequent reduction (9.8 %) in the last three-year period (Fig. 3). Also, the 16^th^ RHO - Apucarana had the most significant relative increase among RHOs, with a 333.0 % increase between 2000 and 2013 (data not shown in the Table or Figure).

## Discussion

The results evidenced that preterm birth rates are growing in Paraná State and vary among the Macro-regional and Regional Health Offices, which reiterates that there is heterogeneity in health indicators in general and in the prematurity rates among different regions, in Brazil and in other low-income countries.

Preterm births lead to serious health consequences, with social and economic impact. Nevertheless, little is known about the inner distribution at the Brazilian states. This study analyzed the trend of preterm births in the state of Paraná. The results indicated that preterm birth rates rose from 6.8 % in 2000 to 10.5 % in 2013, with higher shares of moderate preterm birth (32 to <37 weeks), corroborating the results of other studies [15, 16].

In the United States, prematurity rose from 9.5 % in 1981 to 12.3 % in 2008, leveling at between 12 % and 13 %, whereas in Europe these values went from 5 % to 9 % [16, 17]. In Australia, the rate of preterm births went from 6.8 % in 1991 to 8.2 % in 2009 [18].

Preterm birth is influenced by the degree of socioeconomic development of the population and the location of the study. Nevertheless, one of the possible explanations for the high rates of preterm births and their components lies also in the specificities of the characteristics of mother-child health for each region [19].

In the state of Paraná, in the three-year period from 2011 to 2013, the highest rates of preterm births were concentrated in the RHOs with the highest socioeconomic indicators and Human Development Indexes (HDI), such as the 16^th^ RHO of Apucarana (HDI = 0.79; 11.86 % preterm births), 15^th^ RHO of Maringá (HDI = 0.84; 11.26 %), 17^th^ RHO of Londrina (HDI = 0.82; 11.15 %). The HDI aims to measure the level of development by combining the variables *life expectancy*, *income* and *education*, and ranged from 0 (no human development) to 1 (total human development).

A study analyzing factors related to C-section birth in the state of Paraná detected a link between C-section birth and preterm birth, and also showed an association between this type of delivery and better socioeconomic conditions [20]. Similar results were observed in 2006 for some RHO host cities, which show, respectively, high percentages of C-section birth as well as prematurity rates, with highlight to the municipalities of Maringá (76.1 %; 8.3 %). Umuarama (71.7 %; 9.9 %) and Ivaiporã (66.7 %; 21.4 %) [19].

Brazil has one of the highest rates of C-sections worldwide [21], with a significant increase in recent years. The rate rose from 39.6 % in 2002 to 55.6 % in ten years. In the state of Paraná, these rates are even higher and went from 45.1 % in 2002 to 61.9 % in 2012 [9]. This makes it necessary to question the real need of elective C-sections, their benefits and downsides. Also future studies are required to effectively confirm a possible link between C-section birth and preterm birth in Brazil, as it is known that the high frequency of obstetric interventions is one of the reasons for increased prematurity [16].

The 16^th^ RHO - Apucarana shows the most significant relative increase in moderate preterm birth during the period (333 %), which went from 3.7 % in the 2000–2002 three-year period to 15.9 % in 2011–2013. Thus, that RHO requires greater attention in order to detect the factors associated with this expressive increase, as even newborns between 32 and 36 weeks are immature and have significantly higher rates of morbidity and mortality compared to those born at full term [22].

Deficits in the effectiveness of preventive actions offered to expectant mothers during the prenatal period could partially explain the high percentage of prematurity, given their close link to preterm birth [22]; nevertheless, the percentage of women who have less than seven prenatal visits is low at most RHO host cities in Paraná [19]. Thus, in addition to the number of prenatal visits, the quality of prenatal care must be taken into account, particularly regarding the evidence-based interventions that can possibly make care more efficient. A prenatal care allows for early diagnosis of fetal or maternal problems that may place the fetus in risk, and can therefore prevent complications and incidents that may lead to an early labor [23]. Therefore prenatal care must be even more encompassing among women at greater risk of preterm birth, such as multiple pregnancies and fetuses with congenital malformations [16, 24].

The 7^th^ RHO - Pato Branco was the only one to show a declining trend for prematurity, with a 20.1 % from 2000–2002 to 9.8 % in 2011–2013, due especially to the decrease in the moderate preterm birth component, which fell from 18.8 % to 12.8 % during that period. Some reasons may justify this reduction: the improved Human Development Index of the city, which went from 0.71 in 2000 to 0.78 in 2010, with the expansion in the access of formal education – the aspect that grew the most in absolute terms [11]; as well as greater access to health services, with expanded coverage of the Family Health Strategy (ESF), which rose from 22.6 % in 2000 to 49.8 % in 2013. Nevertheless, directly-linked causes must still be studied further.

Highlight must also be given to the 11^th^ RHO – Campo Mourão, which showed a decrease in preterm birth rates at the start of the period, given the percentage variation of 6.05 % in 2000, 6.77 % in 2001, 7.83 % in 2002, 6.94 % in 2003 and 6.91 % in 2004, falling to 4.88 % in 2005, with a subsequent increase. With regard to the 1^st^ RHO - Paranaguá, 20^th^ RHO - Toledo, 21^st^ RHO - Telêmaco Borba and 22^nd^ RHO – Ivaiporã, there was no statistically significant trend due to the fluctuation in prematurity rates in the study period.

The object of this study was not to identify the factors associated with preterm births in the state of Paraná; nevertheless, it is important to emphasize that improved access to healthcare, with the expansion in ESF coverage statewide, was not capable of reducing the occurrence of preterm births.

In addition to elective C-sections, induced labors and unsatisfactory quality of prenatal care [16, 25], it should also be considered that the rise in preterm births is strongly associated with pregnancies of women 35 or older which frequency has increased. Moreover women over 35 are more susceptible to infertility problems, the treatment of which is linked to higher rates of multiple pregnancies, which are also associated with higher rates of deliveries before 37 weeks of gestation [16].

Another noteworthy result is that although extremely premature (<28 weeks) and very premature (28 to <32 weeks) births have showed low average annual growth rates, their distribution in the thematic maps revealed that the prevalence of extremely premature births doubled at the 15^th^ RHO - Maringá, 17^th^ RHO - Londrina and 18^th^ RHO - Cornélio Procópio, while the rates of very premature births doubled at the 15^th^ RHO - Maringá and 2^nd^ RHO – Metropolitan. It is important to remember that extremely premature and very premature births represent a high risk for severe morbidities, hospital re-admissions, permanent disabilities and mortality, possibly leading to severe economic, social and emotional consequences for the family and society [26, 27].

Some limitations of this study are noteworthy, such as those related to information obtained from secondary databases, especially the possibility of differences in the reliability of data among the different regions. Thus, whenever long time periods are analyzed and different regions are compared (as in this study), any trend in preterm birth rates may be hidden by incorrect entry of gestational age. Also any increase trend could be partly related to gradual improvement in the quality of data entry in declarations of live births.

In addition, the accuracy of the information about gestational age that is registered in the Sinasc database may have discrepancies among the State regions and during the long period of time. Until 2009, the Sinasc database system grouped the gestational age variable in an age strata, only after this year it has been recorded according to exact number of gestational weeks. This made it impossible to analyze borderline prematurity (34 to <37 weeks) and likely induced classification errors, with a tendency towards recorded gestational age ranges closer to full-term births.

Despite these limitations, the data available in Sinasc national system and other databases that have similar health information in Brazil make possible to know and monitor the health situation of the population and identify differences among cities, regions, states and macro-regions. These results allow the evaluation of healthcare policies and interventions, as well as possible inequalities in life, education and healthcare conditions.

## Conclusions

Despite the policies implemented to prevent the preterm birth, our results showed increase of this event in the Southern of Brazil, with differences between Macro-regional and Regional Health Offices. These results confirm what is observed in other countries. However, further studies must be carried out in order to understand the role of socioeconomic and demographic factors, healthcare and organization of health services in the occurrence of preterm births, as well as how risk factors are distributed in different regions and whether the variations in the preterm birth rates can be attributed to local characteristics.

The increase of preterm birth rates shows that measures to prevent this event need to be improved, especially with the moderate preterm births. Births which occurred from 32 to less than 37 gestational weeks are usually related with caesareans, that has extremely high rates in Brazil and in another countries with the same medicalized model of assistance. Therefore, is also important to monitor the rates of unnecessary caesarean and direct the public polices to the regions with the highest rates.

It is worth mentioning the potentiality of use of secondary data provided by the Sinasc. The analysis of variables available in this health system allows to know and monitor the population's health situation, identify differences between the regions, showing results of health care provided in each locality, and possible inequalities in quality of life, education and assistance for the maternal and child health.

## Ethics approval and consent to participate

The research project was approved by the Standing Committee for Ethics in Research of the Paraná State Health Secretariat/Workers Hospital (decision 406,927/2013). All data were obtained from public databases (http://datasus.saude.gov.br/). All data were anonymized.

## Consent for publication

Not applicable.

## Availability of data and materials

The dataset supporting the conclusions of this article is available from public databases in the DATASUS repository (http://datasus.saude.gov.br/).

## Abbreviations

ESF: Family Health Strategy
HDI: Human Development Index
RHO: Regional Health Office
Sinasc: Live Birth Information System
